# Brain Organoids: A Game-Changer for Drug Testing

**DOI:** 10.3390/pharmaceutics16040443

**Published:** 2024-03-22

**Authors:** Chiara Giorgi, Giorgia Lombardozzi, Fabrizio Ammannito, Marta Sofia Scenna, Eleonora Maceroni, Massimiliano Quintiliani, Michele d’Angelo, Annamaria Cimini, Vanessa Castelli

**Affiliations:** Department of Life, Health and Environmental Science, University of L’Aquila, 67100 L’Aquila, Italy; chiara.giorgi2@graduate.univaq.it (C.G.); giorgia.lombardozzi@guest.univaq.it (G.L.); fabrizio.ammannito@student.univaq.it (F.A.); martasofia.scenna@student.univaq.it (M.S.S.); eleonora.maceroni@student.univaq.it (E.M.); massimilianoquintiliani@gmail.com (M.Q.); michele.dangelo@univaq.it (M.d.)

**Keywords:** drug, brain, therapeutic approaches, neurological disorders, Alzheimer’s disease, glioma, Creutzfeldt–Jakob disease, Niemann Pick disease, drug screening

## Abstract

Neurological disorders are the second cause of death and the leading cause of disability worldwide. Unfortunately, no cure exists for these disorders, but the actual therapies are only able to ameliorate people’s quality of life. Thus, there is an urgent need to test potential therapeutic approaches. Brain organoids are a possible valuable tool in the study of the brain, due to their ability to reproduce different brain regions and maturation stages; they can be used also as a tool for disease modelling and target identification of neurological disorders. Recently, brain organoids have been used in drug-screening processes, even if there are several limitations to overcome. This review focuses on the description of brain organoid development and drug-screening processes, discussing the advantages, challenges, and limitations of the use of organoids in modeling neurological diseases. We also highlighted the potential of testing novel therapeutic approaches. Finally, we examine the challenges and future directions to improve the drug-screening process.

## 1. Introduction

Neurological diseases are disorders that affect the peripheral and central nervous system [1]. These kinds of disorders influence the ability of people to walk and speak, and, in general, they can cause significant damage to the cognitive functioning of the brain [1,2]. They are the second leading cause of death and the primary cause of disability worldwide [3]. There are more than 600 diseases related to the nervous system; among them, there are brain tumors; neurodegenerative disorders, such as Parkinson’s disease (PD), Alzheimer’s disease (AD), multiple sclerosis (MS), epilepsy, dementia, and headache disorders; neuroinfections, such as viral infections (i.e., HIV, Zika), bacterial infections (i.e., Neisseria meningitides and Mycobacterium tuberculosis), fungal-related infections (such as Aspergillus and Cryptococcus), and parasitic infections (such as Chagas and malaria); strokes; traumatic brain injuries [4]; and neurodevelopmental disorders, such as Microcephaly and autism spectrum disorders. One of the prevalent environmental risk factors is the increasing aging population; other risk factors could be the population growth, the increased life expectancy, and the increased urbanization [2,5,6]. In addition, the diagnoses of these illnesses is a developing problem worldwide [1]. Another challenge to overcome for the diagnoses of neurological diseases is their heterogeneous and atypical manifestations [7]. Some preventive approaches can be used to improve the quality of life of patients; combining exercise and dietary management seems to be beneficial for neural health promotion that affects the plasticity of the nervous system [8]. Unfortunately, despite the rapid development of interventions from recent years, most patients with neurological disorders are diagnosed late. Another relevant concern regards therapeutic approaches for these diseases. Indeed, the currently available drugs may have adverse side effects [2], and they are also limited due to the poor approval rates [9]. Moreover, there is currently no cure for these diseases, and no effective treatments are able to counteract neurodegeneration, dementia, or to recover injured brains. In recent years, following the above-mentioned reasons, there has been an increasing interest in developing new treatments [10] and new approaches for the study of these diseases. 

To develop new treatments that can be administered to patients, preclinical and clinical studies to assess toxicity must be performed. For this purpose, there are different steps that must be followed in a specified way. Following the screening of the component, toxicity and efficacy tests on in vitro models and also on animal models were performed, and, if the results are promising, there will be clinical studies, followed by the release of the drug on the market [11]. Unfortunately, in the final phases of the clinical trials, a majority of these products are not considered eligible, and some of them fail, even if the preclinical studies provided promising results. One of the reasons for this discrepancy could be due to the differences between experimental animal models and human patients; for example rodents have a lower percentage of white matter than humans [12,13]. Moreover, the animal models are not suitable for high-throughput screening [14]. A possible way to reduce the gap between animal models and human patients could be the use of new in vitro models, such as Organoids [12]. 

**Figure 1:**
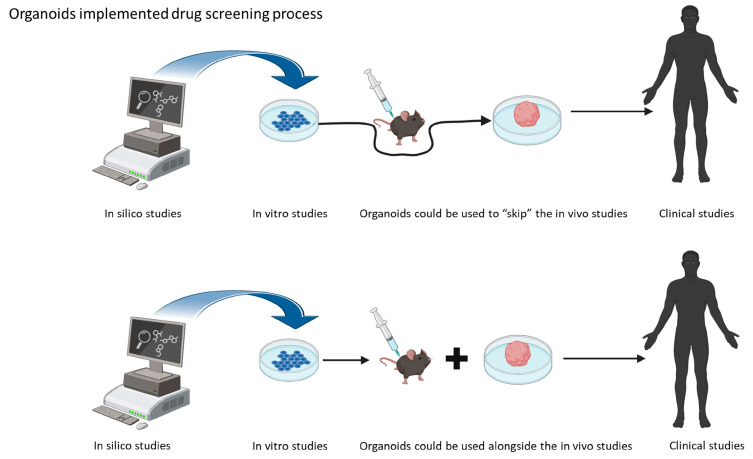
Schematic representation of the possible use of organoids in drug screening research.

Organoids are three-dimensional (3D) cell culture models, deriving from human stem cells. The peculiarity of these 3D models is that they contain cell types that are able to self-organize in a similar way to the organ they are programmed to mimic [15]. These 3D structures mimic the real growth environments of cells under physiological conditions. The use of induced pluripotent stem cells (iPSCs) and their neuronal induction enables the use of patient-derived neurons for the study of disease mechanisms, as well as drug screening [14]. One of the applications of brain organoids is in disease modeling. This means that, with the help of this new in vitro technology, the interspecies differences could be overcome, and the demand for animal facilities could be reduced, accelerating the process of drug screening. As mentioned before, organoids offer an advantage in studying disease in vitro, as they provide a 3D environment that resembles the affected tissue [16] (Figure 1).

In this review, we will focus on brain organoids, beginning with the history, then discussing their potential applications and their use in research as a valuable tool for drug screening in neurological diseases.

## 2. Organoids

### 2.1. History of Organoids

The first attempt at creating and generating a tissue in vitro, marking the beginning of organoid technology, was in 1907, when Wilson demonstrated that sponge cells could self-organize, thus regenerating the organism [17]. Since then, there has been more research demonstrating the ability of disaggregated cells to self-organize and reaggregate; more recently, research groups performed these disaggregation–reaggregation experiments, attempting to generate different organs using amphibian pronephros and chick embryos [18]. However, the meaning of the word has changed with the development of embryonic stem cells (ESCs) and induced pluripotent stem cells (iPSCs) [19]. In 1981, pluripotent stem cells (PSCs) were first isolated from mouse embryos [20], and, in 1998, scientists isolated and cultured embryonic stem cells derived from human blastocysts for the first time [21]. The shift from 2D to 3D started when, in 2008, a research group generated the cerebral cortex tissue from ESCs, using the 3D aggregation culture method [22]. In 2009, the first organoid derived from a single adult stem cell (ASC) was generated; Sato et al. showed that a single leucine-rich repeat containing G protein-coupled receptor 5 (Lgr5)-expressing adult intestinal stem cell could create a 3D intestinal organoid in Matrigel. The cells were able to self-organize and differentiate into crypt-villus structures in the absence of a mesenchymal niche [23]. This work was the starting point for many subsequent organoid works in other systems, such as neuroectoderm (brain and retina), using both ASCs or PSCs [18]. 

Therefore, contemporarily, the term organoid refers to cells grown in vitro in a defined 3D environment to form clusters of cells that can self-organize and differentiate into functional cell types that are then able to recapitulate the structure and function of an organ in vivo. Self-organization within the organoid occurs through cell sorting, which requires the activation of various signaling pathways, mediated by intrinsic cellular components or extrinsic environments, such as an extracellular matrix (ECM) and media [18]. Among some of the different characteristics an organoid should have, there is a 3D structure that contains all the cells present in the model organ, the presence of specialized organ functions, the organ’s self-organization ability, and the presence of different cell types than can be found in the organ [24,25].

### 2.2. Organoids Culture and Modeling

The differentiation protocols needed to form organoids deriving from ESC/iPSC use various growth and inhibitor factors that have a role in the developmental steps of gastrulation and organogenesis. In fact, ESCs and iPSCs have pluripotent properties that enable the generation of all three germ layers (useful for studies of early stage embryonic development), and the capacity of self-renewal and differentiation into different cell types, allowing the organization of cells into an organ-specific pattern (useful for studies of diseases) [18,19]. Another particular aspect of organoids is that they can derive from cells of either patients with diseases or healthy donors. Resulting from cell manipulation, it was possible, for example, to reprogram cells from the fibroblasts of a microcephaly patient into iPSC in order to then compare it to a healthy cell-derived organoid [26]. This was also possible, as it was completed using studies focused on autism, to genetically manipulate cells to form non-idiopathic autism spectrum disorder brain organoids [27].

In more detail, human PSCs, for example, can be induced to undergo differentiation steps that mimic the formation of embryonic germ layers, creating the so-called embryoid bodies (EBs). EBs are 3D cell aggregates that spontaneously form the three germ layers; an enrichment in cell type can be induced via adding molecule additives and specific proteins [28]. To allow the formation of these 3D aggregates, there are different ways and protocols that avoid the direct contact of cells with the plastic support, using both scaffold and scaffold-free techniques. Scaffolds are used to resemble the ECM, and they can be biological or synthetic. One of these is Matrigel, a protein mixture derived from mouse sarcoma cells [18]. For these techniques, organoids are usually cultured in a spinning bioreactor, useful for the promotion of tissue amplification and differentiation [29]. In scaffold-free techniques, an adaptation of the “hanging-drop” cultures, often used to culture multicellular tumor spheroid (MCTS) and also in spheroid formation [18,30], is used. Moreover, to allow the formation of EB, different kind of plates can be used, such as V/U-bottom wells, 3D-printed wells, or low-adherence plates. Some research groups use engineered materials, such as microfilaments and microfluidic chips [31]. It was also demonstrated that it is possible to generate organoids from PSC without the use of ECM [32].

### 2.3. Brain Organoids

Neuronal organoids are very valuable tools in the study of the brain due to their ability to reproduce different brain regions that can interact with each other, or to develop and resemble a specific section of the brain [33,34]. This is possible, even if there is variability among different brain organoids, because they contain the same cell types present in the human brain [35]. In human brain organoids, a typical layer structure is not present, but the different cell subclasses that develop in a brain organoid are organized in a multilaminar fashion. Additionally, they develop in a specific way, making them similar to the human brain [26,29].

The human central nervous system (CNS) is organized following a principle that is typical of all mammals; starting as a neural tube, it later acquires mature organizational features via molecular and cellular processes, forming three main regions: the forebrain, the midbrain, and the hindbrain [36,37]. Cerebral organoids can follow the brain development for 24 weeks after conception; however, after this period of time, organoids start to develop a necrotic core due to the lack of vascularization [38,39]. Oxygenation and nutrient diffusion are important limiting factors in the process of maturation. To overcome these barriers, research groups started to use spinning bioreactors, which also helped to develop a region-specific brain organoid. In addition, to promote neuronal survival and maturation, BDNF (brain-derived neurotrophic factor) was used [29,35,40]. Other promising methods used to avoid the formation of the necrotic core, and, to allow organoid survival, are represented by the transplantation of the organoids into the adult brain of a mouse, or the slicing of organoids that are then left floating in orbital shakers [41,42].

In 2013, Lancaster was able to create 3D cerebral organoids by developing and improving a technique used years before by Watanabe [18]. Watanabe generated forebrain tissues by plating mouse/human EBs in 2D cultures to later transfer them to a 3D aggregation culture; on the other hand, Lancaster directly embedded EBs in Matrigel, allowing the polarization of neuroepithelial buds, which can form different brain regions once in a bioreactor [18,40,43,44]. Furthermore, Watanabe used a serum-free floating culture of EB (SFEB), later improved by Eiraku, who developed a quicker version of SFEB, resulting in cortical organoids with neuronal progenitor cells, neuronal protein expression, and spontaneous neuronal activity [19].

To date, organoids that mimic specific brain regions have been developed; for example, there are the midbrain, the hypothalamus, the blood–brain barrier (BBB), the cerebellum, and the spinal cord. These organoids are useful screening tools for region-specific deficits [14,45,46,47,48]. It has been observed that organoids display both the inhibitory and excitatory synapses with the presence of presynaptic vesicles; this means that the neurons present in organoids have reached maturation. Calcium imaging also showed spontaneous neuronal activity, a signal of neuronal communication [16,35,40,41]. 

Brain organoids can also be used as a tool for disease modelling and the target identification of neurological disorders [49]. To date, different diseases have been studied with organoids, such as Parkinson’s and Alzheimer’s diseases (neurodegenerative disorders), microcephaly, ZIKA virus-induced microcephaly (neurodevelopmental disease), Down syndrome, autism spectrum disorder, and brain tumors [27,40,50,51,52,53,54].

The current protocols for brain organoid cultures do not allow microglia development, but recent research has proposed a model of brain organoids cocultured with primitive-like macrophages, which were generated from the same human-induced pluripotent stem cells (iMac). These macrophages were able to differentiate into microglia-like phenotypes that modulate neuronal progenitor cell differentiation and proliferation, also promoting angiogenesis. This approach could be used to better study early brain development and microglia-derived neurodegenerative diseases [55]. Furthermore, brain organoids have been used to study the effect of severe acute respiratory syndrome coronavirus 2 (SARS-CoV-2) at a neuronal level, while considering the neurological symptoms developed by SARS-CoV-2 patients [56].

### 2.4. Advantages and Limitations of the Use of Organoids

Organoids have several advantages when compared to classical 2D cell cultures and animal models. Organoids can be used to overcome the limitations of 2D cultures, such as poor differentiation into specific cell types. Indeed, one of the main features of organoids is to have the ability to differentiate cells into all the cell types present in the reference organ, such as the brain [57]. Organoids could also be used in place of in vivo models that fail to reproduce certain disorders. At a neurological level, for example, animal models fail to reproduce and replicate all aspects and complexities of the human brain and of human diseases. Additionally, the lifespan of animals used in research affects how they can develop specific age-related diseases, such as Parkinson’s and Alzheimer’s [58]. Furthermore, it has been demonstrated that SFEB organoids derived from iPSCs are characterized by high consistency and reliability [57,59]. Another peculiar aspect that researchers aim to recapitulate is the presence of heterogeneity, which is typical of the clinical conditions of neurological diseases [60]. Moreover, because organoids can be cultured from cells collected from patients, it is possible to model neurological pathologies with cells carrying specific disease mutation, so that the models are as close as possible to the human phenotype [61]. In addition, organoids are great tools to mimic neural networks of a developing human brain, to model human brain development, to investigate disease etiology, to explore molecular pathways, and also to discover new therapies [62,63,64]. Furthermore, working with organoids is relatively easier than working with animal models; organoids can reduce experimental complexity, allowing the study of human development features that may otherwise be difficult to investigate in animal models [11]. Compared to animal and clinical studies, brain organoids are a good model for human brain diseases due to the difficulty and ethical issues surrounding human samples [65]. For instance, post-mortem brain tissue may have undergone irreversible changes during the process of death, thus limiting its utility. Furthermore, there are several guidelines to follow in order to obtain and use human brain tissues [19]. Another advantage of organoid culture is that they maintain genomic stability, which could make them valuable for high-throughput screening [11,66]. 

Although the use of brain organoids has enriched disease modeling and research in general, there are still numerous limitations to overcome. Some of them have already been discussed, such as the lack of vascularity with the consequent necrotic core, and the absence of BBB that could in vivo prevent drugs from entering the brain [67]. There are different possible solutions to these problems; for example, organoids could be engineered to induce ETV2. They could be co-cultured with epithelial cells, and 3D neural constructs with microglia and vasculature could be created. Or, as mentioned before, they could be transplanted into rodents’ brain or sliced [68,69,70]. 

Additionally, organoids have variation, both among themselves and among different groups, in their efficiency of differentiation, morphology, and variability in cell composition [15]. To increase the homogeneity, micro-scaffolds [71] and mini spinning bioreactors “SpinΩ” [29] could be used to produce specific cellular configurations. To avoid and control the variability and unpredictability of organoids, different control groups for each screening batch from isogenic iPSCs can be created, meaning that the interference from the different genetic backgrounds is reduced [16]. 

Another challenge, especially for the study of diseases related to aging, is represented by the presence of fetal neocortex properties in organoids that are associated with a loss of markers, which is typical of aging. To overcome this, some protocols utilize pretreatments with molecules, accelerating maturation [60,72]. 

Furthermore, recently, a research group developed vascularized brain organoids. They generated vessel organoids via mesoderm induction, followed by the induction of vascular progenitors, and then epithelial cells, followed by subsequent treatments with neurotrophic reagents. Later, this research group fused brain organoids with vessel organoids, creating organoids with complex vessels and neurovasculature. This new approach could be useful in future research, even if it requires more studies concerning the lack of blood flow [67].

See Table 1 for a summary of the advantages and disadvantages of brain organoids.

## 3. Drug Screening

As mentioned above, the process of drug discovery and screening is a multi-step process. It starts with the drug design; following this, there are the in vitro tests, and later, the tests move to animal models (pre-clinical). Finally, the clinical tests with the administration of the drugs to the patients take place. Unfortunately, the clinical tests do not always have a positive outcome; this could be due to the differences between the animal model/s currently used and human patients. Organoids could reduce this gap or could at least be implemented into this process in order to avoid futile patient trials. For example, organoids used to test drugs (such as PTC-124 and Ataluren) were successful in non-neurological animal models, but not in human intestinal organoids modeling cystic fibrosis. These results turned out to be accurate in two-phase clinical studies [11,73,74]. However, there are some challenges that need to be overcome regarding the use of organoids in drug screening, such as cellular heterogeneity, the limited scalability, the lack of reproducibility across protocols, and the varying degree of maturity [11].

Furthermore, there are different processes to follow in order to design new drugs: ligand-based, structure-based, and virtual drug designs. Structure-based drug design is performed via using available structural models of the target proteins and searching small molecule libraries. Ligand-based drug design, instead, relies on the knowledge of known molecules binding to the target macromolecule of interest. Virtual drug design relies on the use of computer-assisted drug design and chemical bioinformatics techniques, such as high-throughput docking. These computational methods are reliable tools when accelerating the drug discovery process [75].

Even though organoids are a valuable tool for drug screening, there are still some obstacles to overcome. Some of these obstacles include the heterogenicity of the techniques, the scalability of the organoids, and the maturity stage of the organoids. Heterogeneity refers to both the iPSC lines and the batch-to-batch variation of organoids. Different iPSCs lines have different abilities to generate the cell types observed in their in vivo counterparts; moreover, the different reagents and molecules used and the absence of any standardized protocols cause more variability within the batch of organoids [35,76]. Another problem is represented by the dimension of the organoid; usually for drug screening experiments, the well plates used have between 384 and 1536 wells, but organoids are too large to fit into these plates that are usually essential for the control of the study conditions [11]. Regarding organoid maturity, organoids may lack neuro-endothelial-glial-immune inter-lineage signaling when compared with their in vivo counterparts. They also lack proper vascularization, which could result in necrosis and an impaired sample analysis [11,77].

Automation could be a way to resolve, at least in part, these problems. It has been demonstrated that a liquid-handling robot could help in the automation of the process; this could be used to seed, culture, and fix the organoids. The organoids derived from this process showed a lower variability of size, cell composition, and morphology [11,78]. 

## 4. Neurological Disorders

Neurological disorders, which affect both central and peripheral nervous systems, were estimated to be the cause of 10 million deaths worldwide in 2019 [2]. Organoids are the latest technology for modeling these diseases, as well as for drug screening studies.

### 4.1. Alzheimer’s Disease

Among the different dementias, Alzheimer’s disease (AD) is the most common, affecting more than 40 million individuals worldwide. It can be familial (5%) or sporadic (95%), and its main characteristic is memory deterioration caused by neuronal loss, brain atrophy, and inflammation [3,79,80]. AD is characterized by neuronal and synaptic loss, extracellular beta-amyloid peptide (Aβ) accumulations, and neurofibrillary tangles composed of intra-neuronal abnormally phosphorylated Tau [81]. As mentioned above, using human samples is not always possible due to the ethical limitations, whereas animal models can be used to study familial mutations, but not sporadic ones. In sporadic AD, the major genetic risk factor is the apolipoprotein E (ApoE) ε4 allele [82]. Two-dimensional cell cultures, which lack the interstitial space, do not present the typical extracellular amyloid aggregates [79]. On the other hand, it has been demonstrated that cerebral organoids integrated with genetic mutations can be used to study and model AD. Moreover, organoids could be generated from cells collected from patients [79,83]. Park and collaborators demonstrated that AD cerebral organoids could be a valid tool for drug screening. They had promising results when testing some FDA-approved drugs on the sporadic AD patient-derived cerebral organoids. 

The experimental study was divided into three main steps: the generation of iPSC-derived cerebral organoids (iCOs), the control nodes of disease identification, and suitable FDA-approved drugs selection and testing. Specifically, they first generated iPSC organoids both from normal and sporadic AD (sAD) participants, as well as CRISPR-Cas9 ApoE4 isogenic organoids. To confirm that the iCOs could show pathological phenotypes, they checked the levels of pathogenic proteins, such as Aβ, and phosphorylated tau. As expected, the pathological organoids secreted higher levels of these proteins than the controls; this means that the iCOs developed from sporadic AD patients recapitulate the pathological features of the disease. Approximately 1300 organoids from 11 participants were used for this study, and they were made to be uniform in size and homogeneous in cell composition. Next, they modelled the AD signaling pathways by constructing a signaling network that was later validated and used to identify the control nodes of the disease. For the last step of the study, they applied a mathematical model, considering a network of molecular pathways and relevant genetic factors, to identify several FDA-approved drugs to be tested on the AD organoids. Then, the degree of AD pathogenesis was quantified by the high-content screening (HCS) imaging system. 

To identify the optimal candidate targets for lowering the amount of Aβ and p-tau, Park et al. performed in silico perturbation analysis. The selection of candidate drugs was completed via output node priority selection; the target drug selection was then based on the perturbation analysis and references to a library of FDA-approved drugs. Finally, they excluded some because were they unsuitable candidates based on their drug properties. The selected drugs were then tested for single therapy and for combinational therapy on iCOs. Astaxanthin and Ripasüdil were tested alone, while the combinations were Ibrutinib + Imipramine, Flibanserin + Everolimus, Ripasudil + Flibanserin, Ripasudil + Abemaciclib, and Methanesulfonate. Upon the application of treatment, the levels of Aβ or tau deposition were monitored. As a result, all candidate drugs were proven to be effective in reducing Aβ or tau deposition, and in keeping neuronal cell viability. Moreover, they performed drug screening using the HCS system, thanks to the homogeneity of organoids which resulted from the several quality control steps included [79]. 

### 4.2. Gliomas

Gliomas are primary brain tumors that begin in the brain parenchyma and have histological features similar to normal glial cells. Of these, glioblastoma is the most common tumor in adults. Glial-derived tumors can invade the normal brain; invading the subventricular zone is also often seen in highly invasive glioma tumors. Invasion of the subventricular zone has a poor prognosis and a high recurrence rate [84]. Glioma stem cells (GSCs), like normal stem cells, are capable of self-renewal and differentiation to produce secondary tumors, and they are responsible for the distinctive features of glioma invasion [84,85]. Glioma cell invasion is driven by the cytoskeleton; tumor microtubes (TMs) represent elongated membrane protrusions, which are rich in actin, microtubules, and myosin [86]. To date, there is no cure against glioblastoma, and the current treatments, such as temozolomide (TMZ) and radiotherapy, when not personalized for each patient, are not so effective [87]. In a study, Zhang and collaborators tried to create a combined system, using both murine orthotopic xenografts and cerebral organoids to better model glioma for drug testing [88].

Specifically, Glioma patient-derived cells (GPDCs) were collected and implanted into both mice, to create orthotopic xenograft, and into human cerebral organoids. GPDCs from six patients were each transplanted into one mouse. To monitor the transplantation, magnetic resonance imaging (MRI) was performed on mice eight days after intracranial implantation, and again 80 days post-surgery; organoids were microinjected on day 20. Their orthotopic models transplanted with GPDCs were able to recapitulate the tumor progression and characteristics such as necrosis and aggressiveness. Moreover, they found that in one case, a xenograft implanted with GPDCs from a patient (indicated as GPDC4) diagnosed with grade II presented grade IV symptoms, indicating the ability of the model to mimic disease progression. The patient, treated with TMZ, was tested 18 months after the grade II diagnosis, and the results showed a progression in the disease, with an updated diagnosis to grade IV. Overall, the results they obtained with the xenograft demonstrated that not only are the GPDC orthotopic models able to maintain the pathological characteristics of the parental tumor, but also that they are able to show tumor progression. Zhang and collaborators tested the effect of TMZ on the GPDC-implanted organoids. First, they performed experiments to understand if the effects of TMZ were different between the organoids and the 2D cell cultures, through the use of cytotoxicity assays. They observed that, in all 2D cultures, there was a dose-dependent cell apoptosis/viability when applying the TMZ treatment. In addition, they analyzed the O6-methylguanine-DNA-methyltransferase (MGMT) promoter, a marker for TMZ response. In this study, Zhang and colleagues did not find an association between organoid response to TMZ and MGMT methylation. For example, GPDC4 organoids had low sensitivity to the treatment, even if they displayed the MGMT methylation, while other samples without MGMT methylation were sensitive to TMZ. Moreover, GPDC4 results contrasted to the ones obtained with the 2D cell culture, where TMZ was efficient, while the other samples showed TMZ sensitivity even in organoids. This means that the 2D system lacks the influence of tumor microenvironment (TME), which has an important role in tumor progression and drugs resistance, but also that mutation analysis alone is not enough to evaluate drug efficacy. Therefore, they integrated xenografts and organoids via exosome and RNA sequencing, providing a synchronous monitoring. For the GPDC4 samples, the results highlighted the characteristic glioblastoma aggressiveness, such as TMZ resistance. These results were consistent with both the ones obtained from xenografts, showing grade IV glioblastoma features, and the patient that had poor disease progression, going from a grade II to a grade IV diagnosis. Moreover, Zhang and colleagues demonstrated that the main characteristics of individual GPDC and the responses to TMZ were preserved both in organoids and the xenograft. Another important finding is that this integrated system is a valuable tool for evaluating patient TMZ responses, meaning that it can be used for more personalized drug testing. Furthermore, because GDPC-transplanted-cerebral organoids gave results that were consistent with orthotopic xenografts, they could be used to test treatments faster, with the possibility to also use high-throughput screening [88].

### 4.3. Creutzfeldt-Jakob Disease

Creutzfeldt-Jakob disease (CJD) is a rare, fatal, progressive neurodegenerative disorder; it can be sporadic, genetic, and acquired. Sporadic CJD (sCJD), by far the most common form, representing 85% of cases, is characterized by brain deposition of abnormal prion protein (PrP) aggregates. The prion that causes this disease is the pathological isoform (PrP^Sc^) of a physiological, host-encoded protein, called a cellular prion protein (PrP^C^) [89]. There are different PrP^Sc^ conformations that give rise to prion strains; this phenomenon is important for developing therapeutics, as some of them could be effective against a certain strain only [90]. sCJD consists of a presenile dementia that lasts for a few weeks, with later appearances of ataxia, myoclonia, and pyramidal and extrapyramidal signs [91,92]. sCJD is characterized by brain lesions; PrP^Sc^ deposition is associated with microglial activation. These are the first signs of the disease, followed by spongiform change and synaptic damage; astrocytic gliosis and neuronal loss are the last to develop. Amyloid plaques are present in 10% of cases [91,93]. This neurodegenerative disease is also characterized by a heterogeneous phenotype, resulting in different morphological variants of PrP^Sc^ deposits. The most common ones are synaptic-PrP^Sc^ microdeposits in the cerebral cortex and molecular layer of cerebellum; plaque-like—well-defined, rounded PrP^Sc^ deposits in the cerebral cortex and other area; perivacuolar—associated with vacuoles in the cerebral cortex; and perineuronal—delineating dendrites of neurons in the pyramidal neurons of the hippocampus [91]. It has been demonstrated that microglia are involved in PrP^Sc^ clearance, but may also promote neuroinflammation, neurodegeneration, and disease progression [94]. The vacuole changes are heterogeneous in morphology [93,95]; moreover, PrP^C^ is highly concentrated in presynaptic terminals, and is highly prevalent in structures associated with synaptic plasticity [96], meaning that the loss of normal PrP^C^ functions may contribute to synaptic dysfunction and neuronal loss [91]. 

To date, there are no functional anti-prion drugs, but Groveman and collaborators tried to demonstrate that brain organoids could be used for drug-screening experiments to find a solution to this problem. Notably, they tested the ability of pentosan polysulfate (PPS), an established anti-prion compound, both as a prophylactic drug treatment and a therapeutic drug treatment in sCJD-infected brain organoids in order to evaluate new therapies against prion infections [90]. PPS is known to inhibit prion propagation in 2D cell cultures, and to be effective on mice models, but it fails to cure prion infections in humans due to the difficulty of penetrating the BBB and the subsequent intra-cranial delivery [90,97,98]. For this study, the researchers used different groups of organoids: DMSO sCJD, DMSO normal brain homogenate (NBH), PPS sCJD, and PPS NBH. Since DMSO is known to inhibit PrP^Sc^ formation and to delay the disease in vivo, the authors decided to also include this condition, and to evaluate if the tested molecule was effective in this case. For the prophylactic study, PPS was not toxic to the organoids and did not alter PrP^C^ expression and localization. Organoids were treated with PPS or DMSO for 7 days, then they were inoculated with NBH or sCJD brain homogenates. Inoculates were removed after 7 days, and, on the 14th day following inoculation, the media was completely changed following the removal of the treatments. To monitor the infection, the prion-seeding activity was evaluated, and the organoids were collected up to 120 days post-inoculation. As a result, NBH organoids, as expected, demonstrated no prion-seeding activity, whereas DMSO-treated organoids presented more prion-seeding activity than PPS-treated organoids. Moreover, DMSO-treated organoids showed the sCJD-PrP staining pattern that was not seen in the PPS-treated ones. This means that PPS was able to slow the accumulation of prion-seeding activity and to reduce the deposition of PrP aggregates. 

For the therapeutic approach study, organoids were firstly inoculated with sCJD brain homogenates or NBH for 7 days, and then they were exposed to DMSO (vehicle) or PPS (PPS-T) from day 63 to day 91 following inoculation. Organoids exposed to DMSO showed an increase in seeding activity, while the ones treated with PPS showed a decrease in seeding activity. Furthermore, to verify if these treatments could cure the infection or if it was necessary to use as a continued treatment, some organoids were left without treatment for 28 days. As a result, the DMSO showed a consistent level in seeding activity, and PPS-treated samples showed lower seeding activity than the DMSO-treated organoids. In both the experiments, there were no metabolic changes, meaning that the changes in seeding activity were a consequence of the treatments. Therefore, PPS was able to reduce the level of prion-seeding activity, preventing the deposition of protease-resistant and aggregated PrP. With this study, Groveman and collaborators demonstrated that organoids can be used as a valid model to assess the efficacy of drugs, thus reducing the use of animals in research [90].

### 4.4. Niemann-Pick Type C Disease

Niemann–Pick disease type C (NPC) is a rare progressive childhood neurodegenerative disease. The underlying causes are mutations in either NPC1 (~95%) or NPC2 (~5%) genes, leading to the progressive neurodegeneration of the central nervous system [99,100]. NPC1 is a late endosomal/lysosomal membrane protein, and NPC2 is a soluble lysosomal protein. A deficit of NPC1 affects how cholesterol is moved and balanced in the cells; moreover, NPC1 mutation is associated with neuronal failure, caused by the aberrant accumulation of unesterified cholesterol and multiple sphingolipid species in lysosomes [99,100]. Another aspect of NPC disease is the impaired autophagic fusion. This, along with cholesterol accumulation and lysosomal damage, causes autophagic stress and neuronal death [101]. The dysfunction in the metabolism of cholesterol and other lipids in the body also causes organ damage, and can be fatal [102]. There is currently no cure for NPC, but there are some treatments that aim to hinder the disease progression and improve the quality of life of patients. There is an urgent need to find a valid and reliable model for this disease. 

In a recent study, Lee and researchers tried to model NPC disease with organoids for the first time, and then they tested known drugs to observe if they showed the same effects that were observed in the animal model [100]. 

Specifically, they used induced neural stem cells (iNSCs) to generate organoids. To model the disease, they used iNSCs with NPC1 mutations, generated from fibroblasts of both normal donors and NPC-affected patients. The NPC organoids were generated from two different patient cell lines. NPC organoids at early stages of development had delayed formation of the expanded epithelium, and, on later stages, they were characterized by a smaller size and reduced expansion rates than the wild-type (WT) organoids. Moreover, NPC organoids showed an inhibited neuronal network formation, with a lower neuronal differentiation than WT organoids. Interestingly, the levels of unesterified cholesterol in NPC organoids were higher than in WT ones, and, as expected, the gene expression profile of WT organoids had an increased neuronal differentiation than NPC organoids. Next, they tested two compounds to see if they could rescue the NPC pathological phenotypes. The first one was the valproic acid (VPA), a histone deacetylases inhibitor, which had already been evaluated with an NPC mouse model. The NPC organoids treated with VPA showed an increase in size and pattern formation of the outer layer when compared to non-treated organoids. Moreover, there was an increase in neuronal expressions in treated organoids with respect to non-treated ones. The other compound tested was Hydroxypropyl-β-Cyclodextrin (HPBCD), a cholesterol transporter known for reducing cholesterol accumulation, and for being effective in an NPC animal model. HPBCD was also effective on NPC brain organoids. VPA has the ability to enhance autophagy, and this could be used to treat lysosomal storage diseases. Thanks to VPA treatment, NPC organoids were able to restore the autophagic flux to the level of WT organoids, and, because of this restored autophagic flux, a reduction in the accumulation of cholesterol was observed. Cholesterol transporter-related genes were analyzed in both treated and non-treated NPC organoids to examine cholesterol metabolism. These genes were downregulated in non-treated organoids, and upregulated in treated ones. The results of Lee and collaborators showed that VPA is a valuable drug for the restoring of neuronal differentiation, autophagic flux, and cholesterol homeostasis, meaning that NPC organoids could be used for further studies [100].

## 5. Conclusions and Future Prospective

Brain organoids are three-dimensional structures that mimic the development and function of the human brain in vitro. They have great potential for drug screening, as they can model complex neurological diseases and test the efficacy and toxicity of novel compounds. However, several limitations and challenges need to be addressed before brain organoids can be widely used for this purpose. The most relevant issue is the variability and reproducibility of brain organoid generation and maturation, which can affect the quality and consistency of the results [103,104]. To address this point, the development of standardized protocols and quality control methods for brain organoid generation and characterization is crucial. In addition, the lack of vascularization and BBB in brain organoids, which normally limit the delivery and response of drugs, can also prove problematic. To this end, integrating brain organoids with microfluidic systems, bioreactors, sensors, and other organoids can be helpful in creating more realistic and dynamic models of the human body. Nevertheless, additional work must be completed to incorporate non-neuronal cell types and vasculature, or to find a better solution for the necrotic core of older organoids. Another problem to overcome is the presence of the fetal neocortex, as mentioned before, which is associated with a loss of aging markers. This could alter the study of diseases related to aging. Moreover, organoids, while maintaining an organized structure, still lack the six layers of cytoarchitecture. 

In any case, the efforts that the scientific community is making are remarkable, and are paving the way for more relevant and reliable drug-screening applications using brain organoids.

Certainly, organoids, when compared to classical 2D cultures, show numerous advantages, and could be used to overcome the limitations of animal models, which are not the best option for representing some diseases. Another advantage of patient-derived organoids is the presence of gene mutations which are typical of the disease, allowing researchers to avoid using post-mortem brain samples from patients. 

However, brain organoids, if well developed, could substitute for the animal models, thus allowing researchers to shorten the drug-screening process and to model human neurological diseases more accurately. 

## Figures and Tables

**Table 1:** Summary of the advantages and disadvantages of brain organoids.

Advantages	High consistency and reliability of organoids from serum-free embryoid bodies derived from human iPSCs [57,59]	
	Reflect the heterogeneity of the clinical conditions of neurological diseases [60]	
	Possibility to model neurological pathologies, starting from cells carrying specific disease mutations [61]	
	Models are as close as possible to human developmental pathologies [61]	
	Mimic neural networks of a developing human brain [62]	
	Less ethical issues than in vivo experiments	
	Easier to work with, regarding other patients and animal models [11]	
	Great tools to investigate disease etiology, to explore molecular pathways, and also to discover new therapies [62,63,64]	
Disadvantages	Lack of vascularity [39]	Possible Solution
		Genetically engineered induction of ETV2 [68] Co-culture with epithelial cells [69] Transplantation of human brain organoids into rodent brains [41] Creation of a 3D neural construct with microglia and vasculature [70] Slicing organoids or maintaining them in cultures between an air–liquid interface [42]
	Heterogeneity in size [15]	Usage of the “SpinΩ,” instrument to measure the shear strength of organoids in different brain regions [29]
	Unpredictability and variability [15]	Control group for each screening batch using isogenic iPSCs to help eliminate interference from the different genetic backgrounds [16]
	Brain organoids show properties of fetal neocortex leading to loss of markers associated with aging [72]	Genetically stimulate aging [60]

## Data Availability

Not applicable.
